# Association très rare: fracture de la diaphyse fémorale associée à une fracture de Hoffa homolatérale

**DOI:** 10.11604/pamj.2014.19.63.4973

**Published:** 2014-09-23

**Authors:** Younes Ouchrif, Issam Elouakili

**Affiliations:** 1 Service de Chirurgie Orthopédique CHU de Rabat, Maroc

**Keywords:** Fracture, diaphyse fémorale, fracture de Hoffa, homolatérale, Fracture, femoral shaft, Hoffa fracture, ipsilateral

## Image en medicine

L'association fracture de la diaphyse fémorale et fracture de Hoffa de l'extrémité inférieure du même fémur est très rare, peu de cas sont décrit dans la littérature. Elles surviennent après un traumatisme à haute énergie surtout lors des accidents de la voie publique. Elles touchent par conséquent une population jeune. Notre patient est âgé de 27 ans, il a été victime d'un accident grave en conduisant sa moto. Le diagnostic est évoqué devant l'association d'une déformation de la cuisse et une hémarthrose du genou. L'examen vasculo nerveux doit être systématique par la palpation des pouls distaux et exploration du SPE et SPI. La confirmation diagnostique est faite après un bilan radiologique comprenant une radiographie standard de tout le fémur et des radiographies de face, profil et ¾ du genou éventuellement complétées par une TDM avec reconstruction 3D permettant de mieux visualisé la fracture de Hoffa. Le traitement est chirurgical consistant en enclouage centromédullaire à foyer fermé de la fracture du fémur, associé à un vissage percutané ou à foyer ouvert de la fracture de Hoffa en fonction des possibilités réductionnelles. Le pronostic est dominé par le risque important de raideur du genou.

**Figure 1:**
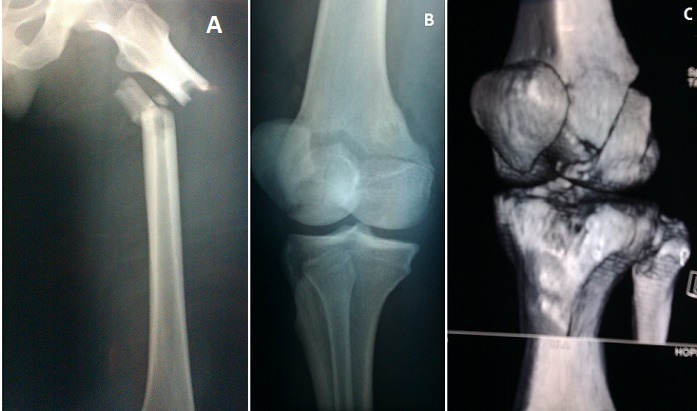
(A) radiographie standard du fémur objectivant une fracture du 1/3 supérieure de la diaphyse fémorale; (B) incidence de ¾ du genou montrant la fracture de Hoffa; (C) TDM avec reconstruction 3D montrant mieux la fracture de Hoffa

